# Four‐hour‐delayed 3D‐FLAIR MRIs in patients with acute unilateral peripheral vestibulopathy

**DOI:** 10.1002/acn3.52123

**Published:** 2024-06-14

**Authors:** Keun‐Tae Kim, Sangeun Park, Sun‐Uk Lee, Euyhyun Park, Byungjun Kim, Byung‐Jo Kim, Ji‐Soo Kim

**Affiliations:** ^1^ Department of Neurology Korea University Medical Center Seoul South Korea; ^2^ Department of Radiology Korea University Medical Center Seoul South Korea; ^3^ Neurotology and Neuro‐ophthalmology Laboratory Korea University Medical Center Seoul South Korea; ^4^ Department of Otorhinolaryngology‐Head and Neck Surgery Korea University Medical Center Seoul South Korea; ^5^ BK21 FOUR Program in Learning Health Systems Korea University Seoul South Korea; ^6^ Dizziness Center, Clinical Neuroscience Center Seoul National University Bundang Hospital Seongnam South Korea; ^7^ Department of Neurology Seoul National University College of Medicine Seoul South Korea

## Abstract

**Objective:**

Conventionally, MRI aids in differentiating acute unilateral peripheral vestibulopathy/vestibular neuritis (AUPV/VN) from mimickers. Meanwhile, the diagnostic utility of MRIs dedicated to the inner ear remains to be elucidated for diagnosing AUPV/VN.

**Methods:**

We prospectively recruited 53 patients with AUPV/VN (mean age ± SD = 60 ± 15 years, 29 men). Initial MRIs were performed with a standard protocol, and an additional axial 3D‐fluid‐attenuated inversion recovery (3D‐FLAIR) sequence was obtained 4 h after intravenous injection of gadoterate meglumine. Abnormal enhancement was defined as a signal intensity that exceeded the mean + 2SD value on the healthy side. The findings of neurotologic evaluation and MRIs were compared.

**Results:**

Overall, the inter‐rater agreement for gadolinium enhancement was 0.886 (Cohen's kappa coefficient). Enhancement was observed in 26 patients (49%), most frequently in the vestibule (*n* = 20), followed by the anterior (*n* = 12), horizontal (HC, *n* = 8), posterior canal (*n* = 5), and superior (*n* = 3) and inferior (*n* = 1) vestibular nerves. In multivariable logistic regression analysis, the enhancement was associated with decreased HC gain in video head‐impulse tests (*p* = 0.036), increased interaural difference in ocular vestibular‐evoked myogenic potentials (*p* = 0.001), and a longer onset‐to‐MRI time span (*p* = 0.024). The sensitivity and specificity were 92.3% and 81.5%, respectively, with an area under the curve of 0.90 for predicting gadolinium enhancement.

**Interpretation:**

Robust gadolinium enhancement was observed on 4‐hour‐delayed 3D‐FLAIR images in nearly half of the patients with AUPV/VN, with a good correlation with the results of neurotologic evaluation. The positivity may be determined by the extent of vestibular deficit, timing of imaging acquisition, and possibly by the underlying etiology causing AUPV/VN. MRIs may aid in delineating the involved structures in AUPV/VN.

## Introduction

Vestibular neuritis (VN) refers to a syndrome of acute unilateral peripheral vestibulopathy (AUPV) due to inflammation of the peripheral vestibular organ.^1^ The diagnosis is centered on clinical features and neurotologic findings when other causes are readily excluded.^2^ Patients invariably suffer from spontaneous vertigo, nausea/vomiting, and unsteadiness. Along with these hallmark symptoms, video‐head impulse tests (video‐HITs) and caloric tests can identify peripheral vestibular hypofunction in the presence of contralesionally beating horizontal‐torsional nystagmus.^2^ In this context, imaging is mostly adopted for differentiation of other causes of vestibulopathy, especially when neurotologic findings are atypical or inconspicuous (i.e., pseudo‐VN). Most importantly, MRIs can differentiate AUPV/VN from posterior circulation strokes, which are potentially dangerous mimickers.

The diagnostic yield of MRIs in AUPV/VN has been sought through earlier studies,^3^ but pathologic finding was not found for the vestibular nerve, ganglion, or labyrinth.^3^ Meanwhile, recent advances in imaging technology have improved the identification of involved structures in various vestibular and auditory disorders. For instance, MRIs can visualize endolymphatic hydrops (EH), a possible radiologically relevant finding of Meniere disease (MD).^4^ Likewise, gadolinium enhancement can be observed in AUPV/VN either at the primary vestibular afferents^5^, ^6^ or in the inner ear.^7^ However, the relationship between neurotologic findings and gadolinium enhancement remains to be elucidated in AUPV/VN. Thus, we investigated the diagnostic yield of 4‐hour‐delayed inner ear imaging in patients with AUPV/VN and determined the association between imaging and neurotological parameters.

## Materials and Methods

### Patients

We prospectively recruited 66 patients with AUPV/VN who underwent neurotologic evaluation and inner ear MRI between September 2019 and January 2023 at Korea University Medical Center. Among them, patients with a history of vestibulopathy that may affect the results of HITs (*n* = 8, three with vestibular migraine, three with chronic otitis media, one with MD, and one with posterior fossa large meningioma) were excluded. Additionally, we excluded patients who showed EH in either the affected or healthy ear (*n* = 5). Even though they had no clinical symptoms compatible with MD, thereby we could rule out the potential influence on the results of neurotologic evaluations. AUPV/VN was diagnosed based on the diagnostic criteria proposed by the Barany Society.^2^

### Neurotologic evaluation

In addition to a standard neurologic examination, all patients had bedside evaluation and video‐oculographic recording of spontaneous (SN), gaze‐evoked (GEN), and head‐shaking nystagmus (HSN; SLVNG, SLMED, Seoul, South Korea).^8^, ^9^, ^10^ HITs were measured in all patients using video‐HITs (SLVNG, SLMED, Seoul, South Korea). Detailed methods for the video‐HITs have been described previously.^10^, ^11^, ^12^

Patients also underwent bithermal caloric tests, subjective visual vertical (SVV; NDI‐150, M2S, Seoul, South Korea) measurements, and cervical (cVEMP) and ocular vestibular‐evoked myogenic potentials (oVEMP) as described previously.^13^, ^14^, ^15^ The interaural differences (IAD) of cVEMP and oVEMP amplitudes were adopted for statistical analysis, which were calculated as ([A_Right_ − A_Left_]/[A_Right_ + A_Left_] × 100%), respectively.

### MRIs

MRI was performed using 3T MRI scanners (Magnetum Skyra, Magnetum Prisma, and Magnetum Vida units, Siemens, Erlangen, Germany) and a receive‐only 64‐channel‐phased array coil, as described previously.^12^ Patients underwent a standard MRI protocol for the internal acoustic canal (IAC) with an additional axial 3D‐fluid‐attenuated inversion recovery (FLAIR) sequence, acquired 4 h after intravenous injection with a standard dose of gadoterate meglumine (0.1 mmol/kg, 0.2 mL/kg; Dotarem®, Guerbet, Roissy, France). The standard IAC MRI protocol consists of diffusion‐weighted, T1‐ and T2‐weighted spin‐echo axial, a heavily T2‐weighted 3D turbo spin‐echo sequence (sampling perfection with application‐optimized contrasts using different flip angle evolutions [SPACE]; Siemens), a heavily T2‐weighted 3D FLAIR sequence, and 3D T1‐weighted contrast‐enhanced imagings (magnetization‐prepared rapid gradient echo [MPRAGE]; Siemens). The 3D FLAIR sequence was acquired with the following parameters: slice thickness, 0.6 mm; TR/TE, 9000/418 ms; inversion time, 2500 ms; number of averages, 2; echo‐train length, 167; flip angle, 120°; matrix, 320 × 214; FOV, 165 × 200 mm. The acquisition time was 6 min 42 s.^12^

**Figure 1:**
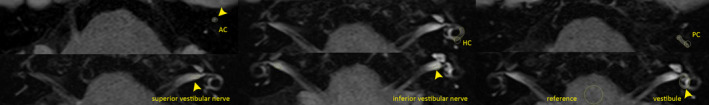
Quantitative evaluation of a degree of the perilymphatic enhancement. Six freehand round or polygonal regions of interests (ROIs) were set manually in AC (3.22–3.68 mm^2^), HC (6.90–9.39 mm^2^), PC (6.90–9.39 mm^2^), canalicular segments of the superior and inferior vestibular nerves (4.60 mm^2^), and vestibule (20.40 mm^2^) on 3D‐FLAIR sequence to include as much of the perilymph as possible. An additional 50 mm^2^ circular ROI was drawn in the medulla at the same plane as the vestibule. The mean signal intensity was recorded for each ROI. The signal intensity ratios of the vestibular nerves and inner ear structure to that of the signal intensity of the medulla were calculated to avoid bias from patient‐related artifacts. AC, anterior canal; HC, horizontal canal; PC, posterior canal.

Six freehand round or polygonal region of interest (ROI) were manually assigned to each neural structure, including the canalicular segment of (1) superior (4.60 mm^2^) and (2) inferior vestibular nerves (4.60 mm^2^), (3) vestibule (20.40 mm^2^), and each semicircular canal for the (4) horizontal (HC; 6.90–9.39 mm^2^), (5) anterior (AC; 3.22–3.68 mm^2^), and (6) posterior canals (PC; 6.90–9.39 mm^2^) independently by two neuroradiologists (B. K. and S. P) with 18 and 2 years of experience, respectively. The signal intensity of the medulla was measured in the same manner as for normalization (Fig. 1). Normalized signal intensity on 4‐hour‐delayed 3D‐FLAIR of the enhancing lesion was defined as the signal intensity of the enhanced portion divided by that of the medulla. Normalized intensities of each organ on the healthy side were used as controls.

The images were independently reviewed by the neuroradiologists who were blinded to the lesion side and clinical characteristics. The signal intensity of each neural structure was calculated using the average value determined independently by neuroradiologists. Later, the signal intensity values were reviewed by the neurologist (S.U.L). And then, abnormal enhancement was defined as when the normalized signal intensities of the affected side exceeded the mean + 2 standard deviation (SD) of the signal intensity of each neural structure derived from the healthy side (upper normal limit <1.49 and <1.62 for the superior and inferior vestibular nerves, respectively; <0.69 for the vestibule; <0.40, <0.61, and <0.63 for the HC, AC, and PC, respectively).

### Statistical analysis

Nominal or independent variables were compared using the chi‐squared or Fisher's exact test, and continuous or independent variables were compared using the Student's *t*‐test and Mann–Whitney *U*‐test. For logistic regression, all variables with a *p* < 0.05 using an age‐ and sex‐matched univariate analysis were included in the multivariable analysis. Variables with *p* < 0.05 were determined to be significant in the multivariable analysis.

Receiver operating characteristic (ROC) curve analysis was conducted to determine the sensitivity and specificity of continuous variables. Sensitivity and specificity were presented as cut‐off values that maximized the Youden Index (sensitivity + [specificity – 1]) and the area under the curve (AUC).

Statistical analyses were performed using the R software package (version 3.4.0; http://www.r‐project.org), and the significance level was set at two‐tailed *p* < 0.05.

## Results

### Clinical characteristics and neurotologic findings

The detailed clinical profiles of the patients are shown in Table 1. Fifty‐three patients were included in the analyses (mean age ± SD = 60 ± 15 years, 29 men). Intravenous or oral corticosteroid treatment was not attempted in any of the patients. Three patients (3 out of 53, 6%) had inferior AUPV/VN. All patients presented with acute spontaneous dizziness/vertigo (*n* = 53), associated with nausea or vomiting (*n* = 47). None of the patients reported new‐onset headaches, tinnitus, ear fullness, or hearing loss at presentation.

**Table 1:** Comparison of the neurotologic findings with respect to gadolinium enhancement.

	Gadolinium enhancement (+) (*n* = 26)	Gadolinium enhancement (−) (*n* = 27)	*p* Value
Age, mean ± SD, years	63 ± 16	57 ± 14	0.103
Sex, women (%)	14 (54)	10 (37)	0.219
Body weight, mean ± SD, kg	70 ± 15	62 ± 13	0.040
Glomerular filtration rate, mean ± SD, mL/min/1.73 m^2^	87.6 ± 24.3	91.7 ± 26.0	0.552
Involved side, right (%)	13 (50)	14 (52)	≈1.000
Onset‐to‐MRI, median (IQR), days	4 (3–6)	4 (2–5)	0.122
SN without fixation, horizontal SPV, median (IQR), °/s	3.4 (1.9–6.2)	2.1 (1.4–6.4)	0.444
Abnormal HIT gain (%)	25 (96)	21 (78)	0.100
VOR gain of HITs (%)			
Horizontal canal, mean ± SD	0.55 ± 0.22	0.73 ± 0.23	0.006
Anterior canal, mean ± SD	0.73 ± 0.24	0.86 ± 0.22	0.058
Posterior canal, mean ± SD	0.94 ± 0.18	0.97 ± 0.16	0.530
Canal paresis (%)	20 (80)	14 (58)	0.100
HSN (%)	20 (77)	19 (70)	0.589
Abnormal cVEMP^a^ (%)	9 (39)	13 (50)	0.445
Abnormal oVEMP^a^ (%)	18 (82)	14 (52)	0.038
Abnormal SVV tilt^a^ (%)	21 (84)	16 (62)	0.116

cVEMP, cervical vestibular‐evoked myogenic potential; HITs, head‐impulse tests; HSN, head‐shaking nystagmus; IQR, interquartile range; oVEMP, ocular VEMP; SD, standard deviation; SPV, slow‐phase velocity; SVV, subjective visual vertical; VOR, vestibulo‐ocular reflex.

^a^ Caloric tests, cVEMP, oVEMP, and SVV were available in 49, 49, 49, and 51 patients, respectively.

### Results of vestibular function tests

Neurotologic findings are summarized in Table 1. All patients showed spontaneous nystagmus without visual fixation, including horizontal‐torsional nystagmus with (*n* = 36) or without vertical components (*n* = 9), pure horizontal (n = 4), pure vertical (*n* = 2), horizontal‐vertical (*n* = 1), and vertical‐torsional (*n* = 1). The slow‐phase velocity of spontaneous nystagmus ranged from 1.7 to 16.5 (median [interquartile range, IQR] = 3.0 [1.5–6.7]). None of the patients had GEN during lateral gazes. Horizontal head shaking elicited nystagmus in 39 patients (39 out of 53, 74%), the directions of which were mostly contralesional (*n* = 37). Vibration‐induced nystagmus was documented in 33 patients (33 out of 53, 63%) and was almost always beating contralesionally (*n* = 32).

Forty‐six patients (46 out of 53, 87%) showed decreased VOR gain in at least one canal: HC (*n* = 44), AC (*n* = 21), and PC (*n* = 6). Among those with normal VOR gain, the diagnosis of VN/AUPV was determined based on canal paresis >25%. Canal paresis was observed on the lesioned side in 34 patients (34 out of 49, 69%). oVEMP was abnormal in 32 patients (32 out of 49, 65%), showing absent (*n* = 19), decreased (*n* = 11), or delayed responses on the affected side (*n* = 4, including two showing both delayed and decreased responses). cVEMP was abnormal in 22 (22 out of 49, 45%) patients, showing delayed (*n* = 11), decreased (*n* = 10, including six showing both delayed and decreased responses), or absent responses (*n* = 4) during stimulation in the affected ear. Two patients showed decreased responses on both sides and one on the healthy side. SVV was tilted in 37 patients (37 out of 51, 73%), always to the ipsilesional side.

### MRI findings

**Figure 2:**
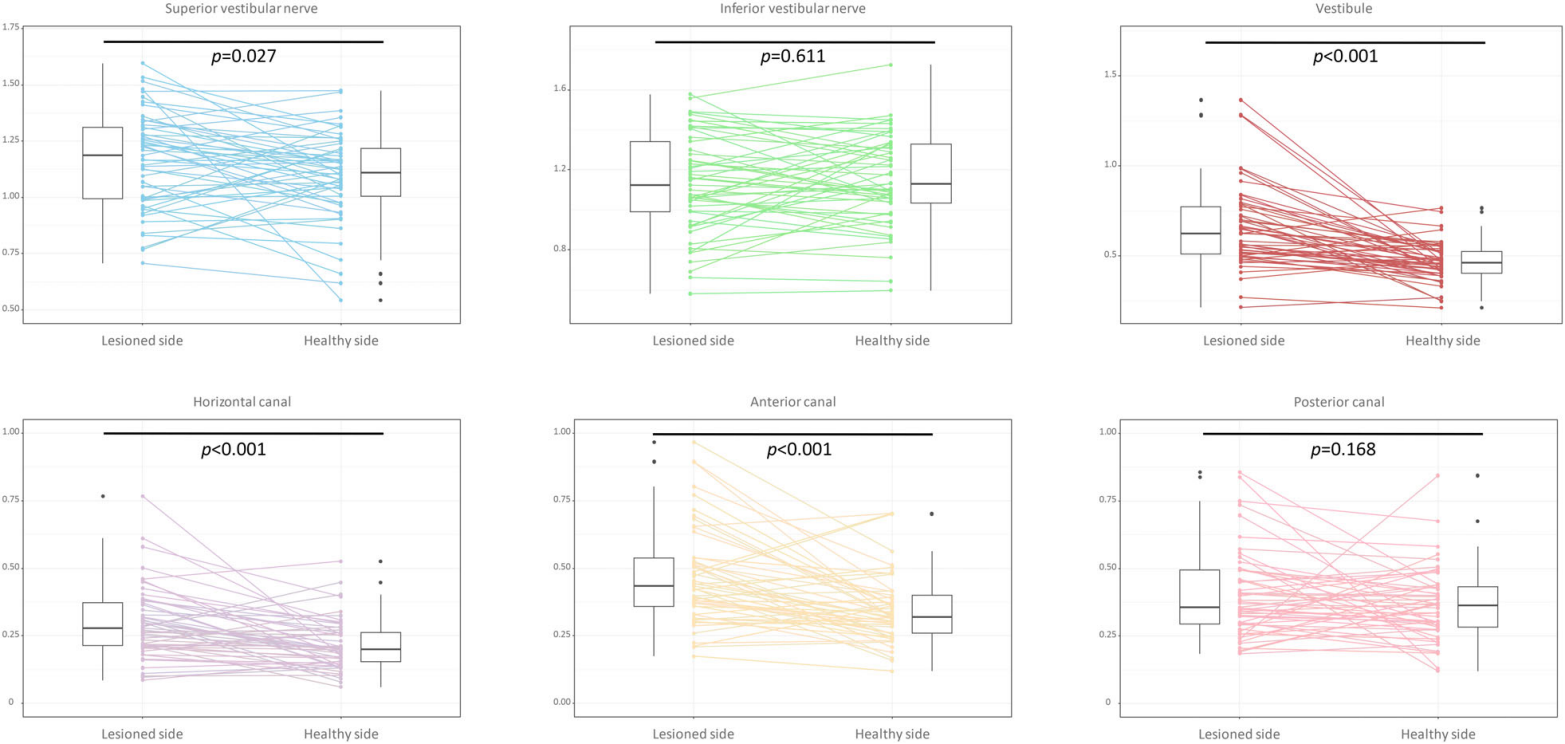
Comparison of gadolinium enhancement of each neural structure between the sides. Contrast enhancement was more prominent in the superior vestibular nerve (1.16 ± 0.21 vs. 1.09 ± 0.20, *p* = 0.027), vestibule (0.66 ± 0.23 vs. 0.47 ± 0.11, *p* < 0.001), HC (0.30 ± 0.13 vs. 0.22 ± 0.09, *p* < 0.001), and AC (0.48 ± 0.21 vs. 0.35 ± 0.13, *p* < 0.001) on the lesioned side than those on the healthy side (Fig. 2). However, no differences were found for the inferior vestibular nerve (1.14 ± 0.27 vs. 1.16 ± 0.23, *p* = 0.611) and PC (0.40 ± 0.16 vs. 0.36 ± 0.13, *p* = 0.168) between the sides.

**Figure 3:**
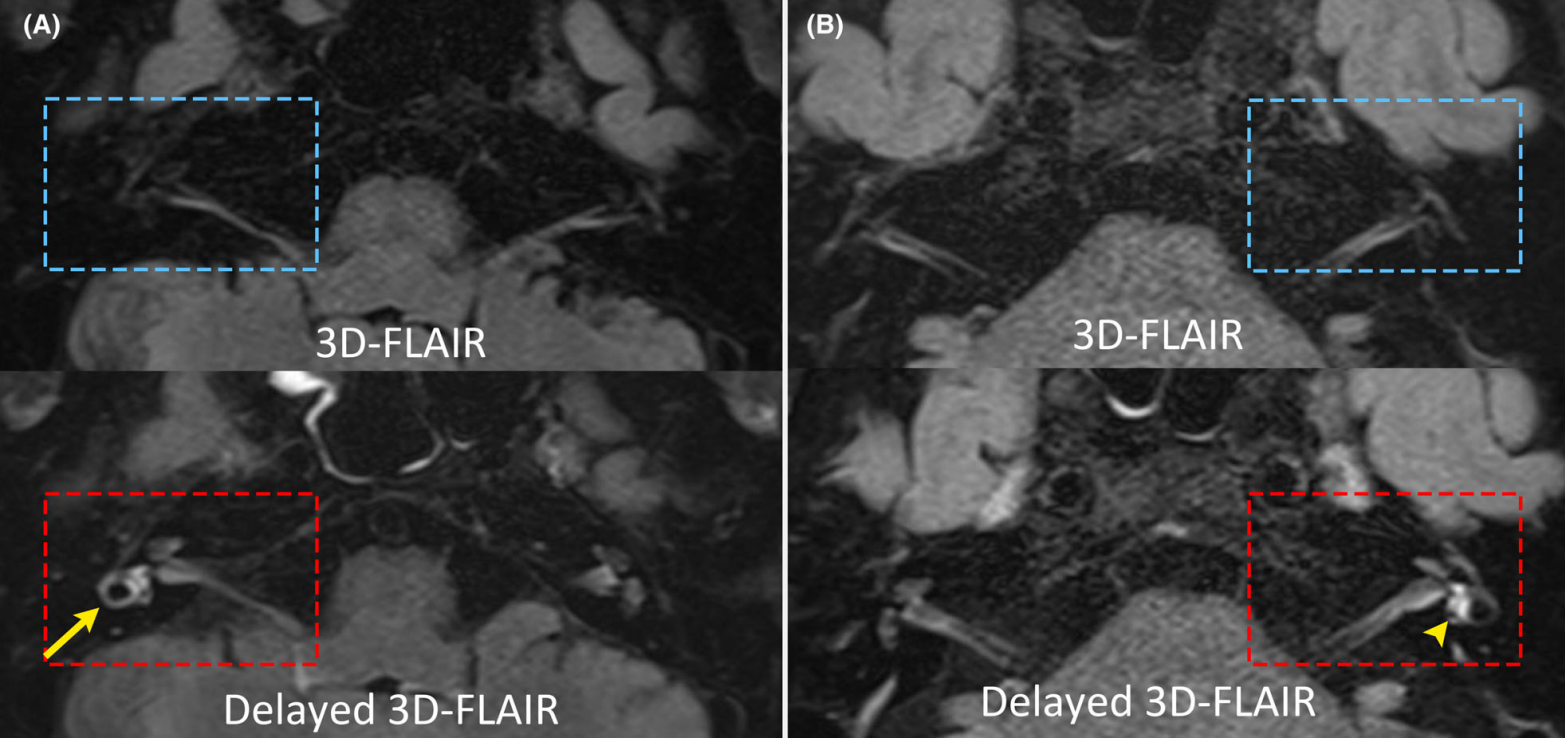
Representative MRIs in patients with acute unilateral peripheral vestibulopathy/vestibular neuritis. (A) No signal changes are present on baseline 3D‐FLAIR image in a patient with right acute unilateral peripheral vestibulopathy/vestibular neuritis (upper row). Contrastingly, a robust gadolinium enhancement is observed in the horizontal canal (arrow) and vestibule on the 4‐hour‐delayed 3D‐FLAIR image (red rectangle; lower row). (B) gadolinium enhancement is found in the vestibule (arrowhead) on the 4‐hour‐delayed 3D‐FLAIR image (lower row) while showing no discernible changes on baseline 3D‐FLAIR image (upper row).

1. *Inter‐rater correlation*. The overall interrater agreement for gadolinium enhancement (no enhancement vs. positive enhancement) was 0.886 (Cohen's kappa). The kappa values for each neural structure were 0.542 and 1.000 for the superior and inferior vestibular nerves, respectively, and 0.836, 0.680, 0.886, and 0.899 for the vestibule, HC, AC, and PC, respectively.
2. *Affected versus healthy side*. Contrast enhancement was more prominent in the superior vestibular nerve (1.16 ± 0.21 vs. 1.09 ± 0.20, p = 0.027), vestibule (0.66 ± 0.23 vs. 0.47 ± 0.11, *p* < 0.001), HC (0.30 ± 0.13 vs. 0.22 ± 0.09, *p* < 0.001), and AC (0.48 ± 0.21 vs. 0.35 ± 0.13, *p* < 0.001) on the ipsilesional than those on the contralesional side (Fig. 2). However, no differences were found for the inferior vestibular nerve (1.14 ± 0.27 vs. 1.16 ± 0.23, *p* = 0.611) and PC (0.40 ± 0.16 vs. 0.36 ± 0.13, *p* = 0.168) between the sides.
3. *Distribution of gadolinium enhancement*. Twenty‐six patients (26 out of 53, 49%) showed gadolinium enhancement either at the labyrinth (i.e., the vestibule and each semicircular canal) or at the vestibular nerve (superior or inferior). Enhancement was observed most frequently in the vestibule (*n* = 20), followed by the AC (*n* = 12), HC (*n* = 8), PC (*n* = 5), superior (*n* = 3), and inferior vestibular nerves (*n* = 1; Fig. 3).

### Prediction of gadolinium enhancement

Multivariable logistic regression showed that gadolinium enhancement was associated with decreased HC gain (odds ratio [OR] 95% confidence interval [CI] = 0.02 [0.001–0.78], *p* = 0.036; Table 2), greater IAD of oVEMP (1.04 [1.02–1.07], *p* = 0.001), and longer onset‐to‐MRI time span (1.86 [1.09–3.18], *p* = 0.024; Table 3). Contrastingly, the AC and PC gains and the IAD of cVEMP were not associated with gadolinium enhancement.

**Table 2:** Prediction of gadolinium enhancement in overall inner ear and vestibular nerves in patients with AUPV/VN^a^

	Unadjusted OR (95% CI)	Multivariable analysis (95% CI)	*p* Value for multivariable analysis
Age, years	1.03 (0.99–1.07)	1.03 (0.96–1.11)	0.404
Female sex	0.50 (0.17–1.51)	1.63 (0.24–11.19)	0.618
Body weight, kg	0.96 (0.91–1.00)	0.95 (0.88–1.03)	0.220
Glomerular filtration rate, mL/min/1.73 m^2^	1.00 (0.97–1.02)	1.01 (0.97–1.05)	0.637
Onset‐to‐MRIs, days	1.27 (0.97–1.65)	1.48 (0.99–2.22)	0.058
HC gain	0.03 (0.002–0.43)	0.02 (0.001–0.78)	0.036
AC gain	0.09 (0.01–1.14)	0.16 (0.01–4.11)	0.269
PC gain	0.35 (0.01–8.78)	2.06 (0.03–158.07)	0.744

AC, anterior canal; AUPV, acute unilateral peripheral vestibulopathy; CI, confidence interval; HC, horizontal canal; OR, odds ratio; PC, posterior canal; VN, vestibular neuritis.

^a^ The interaural difference of cervical and ocular vestibular‐evoked myogenic potential and VOR gain for each canal were calculated separately for multivariable logistic regression due to multicollinearity.

**Table 3:** Prediction of gadolinium enhancement in overall inner ear and vestibular nerves in patients with AUPV/VN.

	Unadjusted OR (95% CI)	Multivariable analysis (95% CI)	*p* Value for multivariable analysis
Age, years	1.03 (0.99–1.07)	1.02 (0.95–1.09)	0.634
Female sex	0.50 (0.17–1.51)	2.60 (0.21–31.97)	0.456
Body weight, kg	0.96 (0.91–1.00)	0.94 (0.85–1.03)	0.194
Glomerular filtration rate, mL/min/1.73 m^2^	1.00 (0.97–1.02)	1.02 (0.97–1.07)	0.423
Onset‐to‐MRIs, days	1.27 (0.97–1.65)	1.86 (1.09–3.18)	0.024
IAD of oVEMP, %	1.02 (1.01–1.04)	1.04 (1.02–1.07)	0.001
IAD of cVEMP, %	0.99 (0.97–1.01)	0.98 (0.95–1.01)	0.198

AUPV, acute unilateral peripheral vestibulopathy; CI, confidence interval; cVEMP, cervical vestibular‐evoked myogenic potential; IAD, interaural difference; OR, odds ratio; oVEMP, ocular VEMP; VN, vestibular neuritis.

### ROC analysis

**Figure 4:**
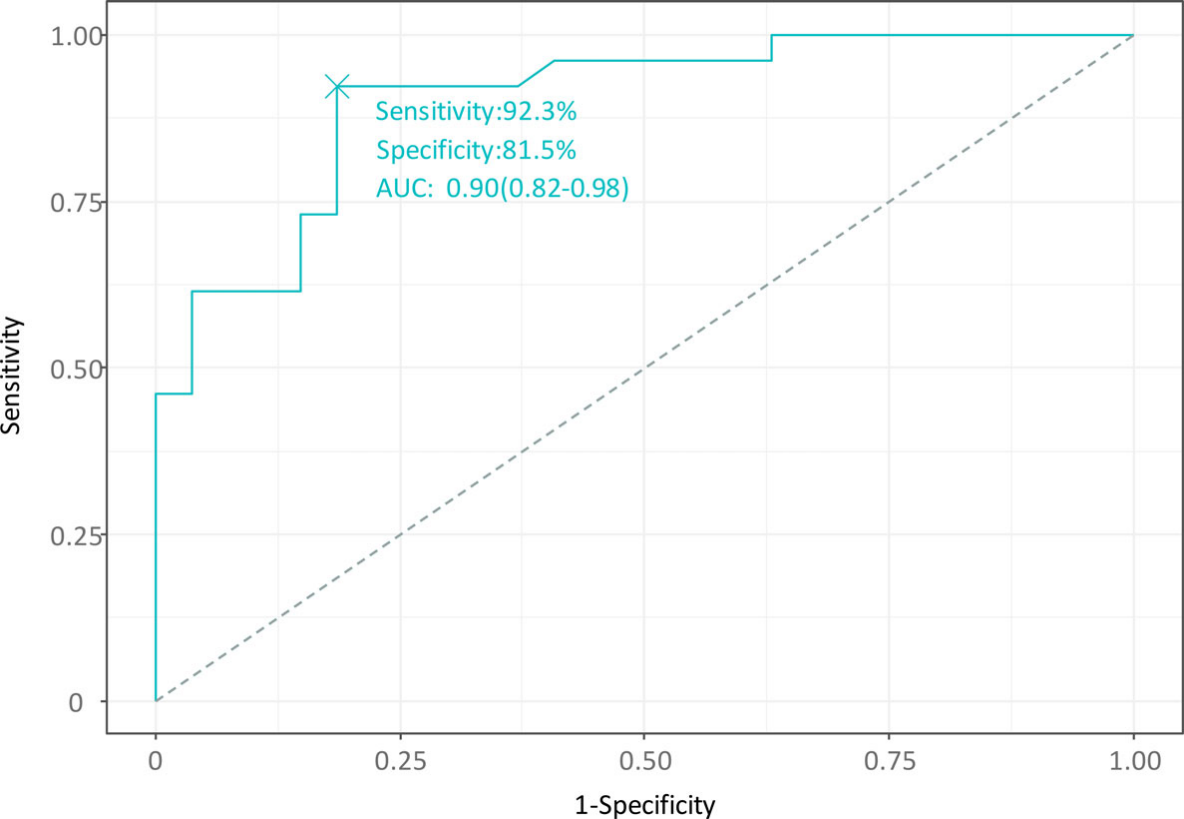
Receiver operating characteristic analysis for VOR gain, IAD of oVEMP, and onset‐to‐MRI for separating gadolinium enhancement from no enhancement on MRIs. The sensitivity was 92.3%, and specificity was 81.5% at cutoff values of 0.95 for HC gain, 100% for IAD of oVEMP, and 4 days for a time span from symptom onset to MRI with an AUC of 0.90 [95% CI = 0.82–0.98]. AUC, area under the curve; HC, horizontal canal; IAD, interaural difference; oVEMP, ocular vestibular‐evoked myogenic potentials; VOR, vestibulo‐ocular reflex.

The sensitivity was 92.3%, and specificity was 81.5% at cutoff values of 0.95 for HC gain, 100% for IAD of oVEMP, and 4 days for the time span from symptom onset to MRI with an AUC of 0.90 (95% CI = 0.82–0.98; Fig. 4).

## Discussion

The main findings of our study can be summarized as follows: 1) Abnormal enhancement was observed in 49% of patients with AUPV/VN. 2) Gadolinium enhancement was observed primarily in the vestibule, followed by the AC and HC. 3) Gadolinium enhancement was associated with the degree of vestibular deficit; a robust enhancement was observed in each corresponding neural structure, showing a profound HC gain decrement and a greater IAD of oVEMP. 4) Together, HC gain <0.90, absent oVEMP response, and time span from symptom onset to MRI more than 4 days predicted gadolinium enhancement with a sensitivity of 92.3%, specificity of 81.5%, and AUC of 0.90.

### Prior reports on MRIs as a diagnostic test for AUPV/VN

Conventionally, the diagnostic yield of brain MRI is limited to differentiating AUPV/VN from central lesions. Bell's palsy has a similar mechanism to AUPV/VN, but it exhibits frequent gadolinium enhancement in the facial nerve,^16^ whereas enhancement has scarcely been observed in AUPV/VN.^5^ This may be due to the small diameter of the vasculature distributed along the vestibular nerve required to show remarkable enhancement from disrupted blood–labyrinthine barrier (BLB).^3^ Additionally, the vestibular damage may be subtle and fail to reach the threshold at which it becomes visible.^3^ Earlier studies investigated the same research question using conventional MRI method, but yielded negative results.^3^

Recently, MRIs targeted at the inner ear have revealed that enhancement can be observed when scanning is performed 1–4 h after gadolinium injection.^7^, ^17^, ^18^, ^19^ Our result suggests that AUPV/VN cannot visualized when the MRIs were conducted too early. Rather, MRIs can show positivity usually after 4 days after symptom onset when AUPV/VN is suspected. It is widely accepted that the timing of the imaging acquisition is important. Yet, the dosage of gadolinium is at odds. The standard dose and higher dose (0.1 vs. 0.15 vs. 0.2 mmol/kg body weight) allowed for the same differentiating power for visualization of endolymphatic spaces.^19^ By contrast, other researchers reported gadolinium might be a factor for positivity,^5^, ^7^, ^20^ as a higher dose (≥0.3 mmol/kg of body weight) has a higher chance of detecting enhancement.^5^ Our study showed that inner ear structures can be readily visualized by using a standard dose of 0.1 mmol/kg of gadolinium. Similarly, gadolinium enhancement can be found in up to 60–69% of AUPV/VN using delayed MR imaging when other factors are adequately controlled.^7^, ^12^ The discrepant results observed across studies may be attributed to differences in the dosage of gadolinium or MRI protocols. Additionally, the method of signal normalization (such as signal‐to‐noise ratio vs. adopting reference ROI) or the definition of ROIs may be another factor to be considered.

### Distribution of the gadolinium‐enhancement and anatomical site of damage in AUPV/VN

Questions are increasingly being raised regarding the precise anatomical lesions in AUPV/VN. It is still unclear whether the vestibular nerve (i.e., neuritis) or vestibular organ (intralabyrinthine) causes AUPV/VN.^21^ Some electrophysiological studies may provide clues. For instance, VEMP responses can be impaired by galvanic and sound stimuli in patients with AUPV/VN. Among these, 27% of patients show abnormal VEMP only on sound stimuli, indicating that a subset of patients may have a selective labyrinthine lesion.^22^ However, prior MRI studies have shown contradictory results: gadolinium enhancement can be found both at the vestibular nerve and the labyrinth.^7^, ^12^ Notably, gadolinium enhancement can be confined to the labyrinth in some patients.^23^ We observed a robust association between neurotological test results and gadolinium enhancement, mostly in the labyrinth. Based on our findings, we assume that both the inner ear and nerves can be involved, but the predilection site for damage may be the labyrinth. Therefore, AUPV/VN may be a limited form of labyrinthine inflammation that spares the cochlea.

Gadolinium enhancement was readily found in AC and HC than in PC. The canal of the SVN is seven times longer and has more spiculae than the canal of the IVN.^24^ Thus, the SVN and its supplying arteriole travel through a relatively narrower passage compared to IVN. In addition, PC may be anatomically resilient to inflammation, as it has double innervation of the two nerves, namely the posterior ampullary nerve and the accessory posterior ampullary nerve, running through a separate bony canal.^25^, ^26^ The wide range of kappa values on the vestibular nerve (0.542 for SVN and 1.000 for IVN) may result from the rarity of enhancement in these structures. It also implies that the inner ear would be suitable for detecting gadolinium enhancement in AUPV/VN.

However, our results should be interpreted with caution, given that AUPV/VN is clinically diagnosed, and various etiologies are inferred as the underlying mechanism. For example, the viral etiology is hypothesized by the reactivation of type 1 herpes simplex virus type I (HSV‐1) in the vestibular ganglia.^25^, ^27^ Temporal bone studies mostly indicate that microvascular occlusion and hypoperfusion of the vestibular nerve or organ is an alternative hypothesis.^28^ Immune‐mediated dysregulation may also play a role in the pathogenesis.^12^, ^29^, ^30^ The damaged site may vary depending on the etiology of AUPV/VN, and a single unifying theory of nerve versus vestibular organ can be misleading. This various etiology causing AUPV/VN may be a factor for determining positivity on MRI. Additionally, 4‐hour‐delayed 3D FLAIR may be more suitable for anatomic discrimination of the inner ear than the nerve, precluding a comparative discussion of the nerve and vestibular organ.^31^ Furthermore, gadolinium enhancement tended to be preferentially found in the HC and AC, omitting the PC. This selective involvement supports the hypothesis that the inflammatory process originates from the vestibular nerve or ganglion.^32^

### Factors as determinants of gadolinium enhancement

Gadolinium enhancement results from a breakdown of either the blood–nerve barrier (BNB) or BLB, or hyperemia of the perineural arteriovenous structure.^33^ Prior MRI studies have pointed out that positivity is associated with persistent spontaneous nystagmus^7^ or the timing of imaging acquisition (onset‐to‐MRI).^23^ This implies that the extent of vestibular damage and the subsequent compensation may be a factor for positivity. However, possible covariates were not statistically controlled for in previous studies.^7^, ^23^ Our findings suggest that the MRI can yield negative results, if the damage is marginal to the threshold to be visible. In addition, current imaging techniques cannot discriminate ischemic injury confined to the labyrinth from inflammation. Future studies on a larger scale can clarify other factors that may influence enhancement in AUPV/VN, other than the extent of vestibular injury or underlying etiology causing AUPV/VN.^12^, ^29^, ^30^, ^34^

Our results align with those of previous reports, showing that the onset‐to‐MRI time can be a factor for gadolinium enhancement. In fact, temporal changes in BNB and BLB disruption have not been fully delineated. The bimodal temporal profile of the blood–brain barrier (BBB) may provide some insight. BBB breakdown proceeds until recovery starts, and a delayed second phase of BBB disruption occurs 3–7 days after a stroke or traumatic brain injury.^35^, ^36^, ^37^ The endothelial caveolae increase in the acute early opening, whereas the tight junction remodeling begins later at delayed time points of ischemic reperfusion injury, exhibiting biphasic temporal changes.^38^ Our results imply that the BNB or BLB breakdown may be analogous to the BBB breakdown.

### BNB and BLB: Anatomical barriers that demarcate the blood–nerve and blood–labyrinth barriers

The BNB is a physiological boundary between the peripheral nerve axons and the bloodstream that prevents the transfer of substances from the plasma to the nerve fibers.^39^ The BNB normally maintains and guarantees axonal function in the peripheral nerves. Similarly, the BLB outlines the impermeable endolymphatic compartment and is selectively permeable to solutes within the endothelial cells of the endoneurial continuous capillaries and in the internal layers of the perineurium.^40^ Endothelial cells from endoneurial vessels are joined tightly together by specialized junctions, minimizing capillary permeability. Adjacent to the capillaries, the perineurial sheath comprises large numbers of tight junctions between perineurial cells, isolating each fascicle from the interfascicular and epineurial environments. When a nerve or labyrinth is damaged, it swells to disrupt the BNB and BLB, which is documented as gadolinium enhancement on MRIs.

### Clinical implication and caveats for future study

Our results should be interpreted with caution, as the diagnostic yield of routine screening of inner ear MRIs in AUPV/VN may be limited. Only nearly half of patients showed positivity, and clinical diagnosis of VN/AUPV is already secured based on clinical and neurotological findings. However, our findings suggest that MRI may aid in AUPV/VN diagnosis, potentially expanding the current clinical spectrum of AUPV/VN. Given the various etiologies that should be considered for causing AUPV/VN,^12^, ^25^, ^27^, ^28^ imaging may aid in stratifying the subtypes according to various etiologies and anatomical sites involved.

Our study has some limitations. Most of all, the lack of a control group limits the validity of our findings. By only comparing the (clinically) affected side with the healthy side, potential physiological side differences in contrast agent uptake pose a relevant limitation. In addition, gadolinium enhancement is not pathognomonic to AUPV/VN and can be found in several other diseases involving the vestibular nerve or labyrinth, such as MD,^41^ labyrinthitis,^42^ vestibular schwannoma,^43^ and labyrinthine ischemia/hemorrhage.^17^, ^44^ Similarly, gadolinium enhancement of other cranial nerves (e.g., the oculomotor or abducens nerves) does not correlate with the clinical manifestation or underlying etiology.^45^, ^46^ Gadolinium enhancement merely reflects the extent of vestibular damage (i.e., disruption of the BNB and BLB).^35^, ^36^, ^37^ Gadolinium enhancement, per se, should not be recognized as a pathognomonic imaging finding of AUPV/VN, and it should be interpreted in consideration of clinical manifestations and neurotologic findings. Furthermore, vestibular impairment can evolve over time. For instance, canal paresis evolves over time, and cases with negative initial results can show canal paresis later.^47^ Thus, the interaction between the nature of vestibular damage and the timing of image acquisition may be a factor to be considered for positivity.

## Author Contributions

K.T. Kim analyzed and interpreted the data and wrote the manuscript. S. Park, E. Park, B. Kim, and J.S. Kim analyzed and interpreted the data, and revised the manuscript. S.U. Lee and B. Kim designed and conceptualized the study, interpreted the data, and revised the manuscript.

## Conflicts of Interest

Drs. K.T. Kim, S. Park, E. Park, and Byungjun Kim report no disclosures. Byung‐Jo Kim serves as an Editor‐in‐Chief of the *Journal of Clinical Neurology*. J.S. Kim serves as an Associate Editor of *Frontiers in Neuro‐otology* and on the editorial boards of the *Journal of Clinical Neurology, Frontiers in Neuro‐ophthalmology, Journal of Neuro‐ophthalmology, Journal of Vestibular Research, Journal of Neurology, Medicine*, and *Clinical and Translational Neuroscience*.
